# The Ion-Translocating NrfD-Like Subunit of Energy-Transducing Membrane Complexes

**DOI:** 10.3389/fchem.2021.663706

**Published:** 2021-04-13

**Authors:** Filipa Calisto, Manuela M. Pereira

**Affiliations:** ^1^Instituto de Tecnologia Química e Biológica-António Xavier, Universidade Nova de Lisboa, Oeiras, Portugal; ^2^BioISI–Biosystems & Integrative Sciences Institute, Faculdade de Ciências, Universdade de Lisboa, Lisboa, Portugal

**Keywords:** NrfD-like, membrane protein, ion translocation, quinine/quinol binding site, CISM family, energy transduction

## Abstract

Several energy-transducing microbial enzymes have their peripheral subunits connected to the membrane through an integral membrane protein, that interacts with quinones but does not have redox cofactors, the so-called NrfD-like subunit. The periplasmic nitrite reductase (NrfABCD) was the first complex recognized to have a membrane subunit with these characteristics and consequently provided the family's name: NrfD. Sequence analyses indicate that NrfD homologs are present in many diverse enzymes, such as polysulfide reductase (PsrABC), respiratory alternative complex III (ACIII), dimethyl sulfoxide (DMSO) reductase (DmsABC), tetrathionate reductase (TtrABC), sulfur reductase complex (SreABC), sulfite dehydrogenase (SoeABC), quinone reductase complex (QrcABCD), nine-heme cytochrome complex (NhcABCD), group-2 [NiFe] hydrogenase (Hyd-2), dissimilatory sulfite-reductase complex (DsrMKJOP), arsenate reductase (ArrC) and multiheme cytochrome *c* sulfite reductase (MccACD). The molecular structure of ACIII subunit C (ActC) and Psr subunit C (PsrC), NrfD-like subunits, revealed the existence of ion-conducting pathways. We performed thorough primary structural analyses and built structural models of the NrfD-like subunits. We observed that all these subunits are constituted by two structural repeats composed of four-helix bundles, possibly harboring ion-conducting pathways and containing a quinone/quinol binding site. NrfD-like subunits may be the ion-pumping module of several enzymes. Our data impact on the discussion of functional implications of the NrfD-like subunit-containing complexes, namely in their ability to transduce energy.

## Introduction

All living organisms need energy to fuel life processes. External energy sources, light or chemical compounds, are converted to biologically usable forms of energy, such as adenosine triphosphate (ATP) or electrochemical gradients. According to Peter Mitchell's chemiosmotic hypothesis, the transmembrane difference of the electrochemical potential ($\Delta \tilde{\mu }$) can be established by energy-transducing membrane protein complexes that couple the energy released by light or chemical reactions (Gibbs energy change, Δ*G*) to the translocation of charges (electrons or ions) across the membrane (Mitchell, 1961). The energy stored in the form of the electrochemical potential can drive different energy-requiring reactions of the cells, such as synthesis of cellular components, solute transport or motility.

Energy transducing membrane complexes are usually composed of catalytic subunits and transmembrane proteins that perform translocation of charges, electrons or cations, across the membrane (Calisto et al., 2021). The most common membrane charge-translocating subunits so far observed in energy transduction complexes are the di-heme cytochrome *b*-like subunits and the so called NrfD-like subunits. The di-heme cytochrome *b*-like subunits are involved in the transport of electrons, whereas the NrfD-like subunits translocate ions. In this way this type of subunits is devoid of redox cofactors but contain ion-conducting pathways.

**Figure 1:**
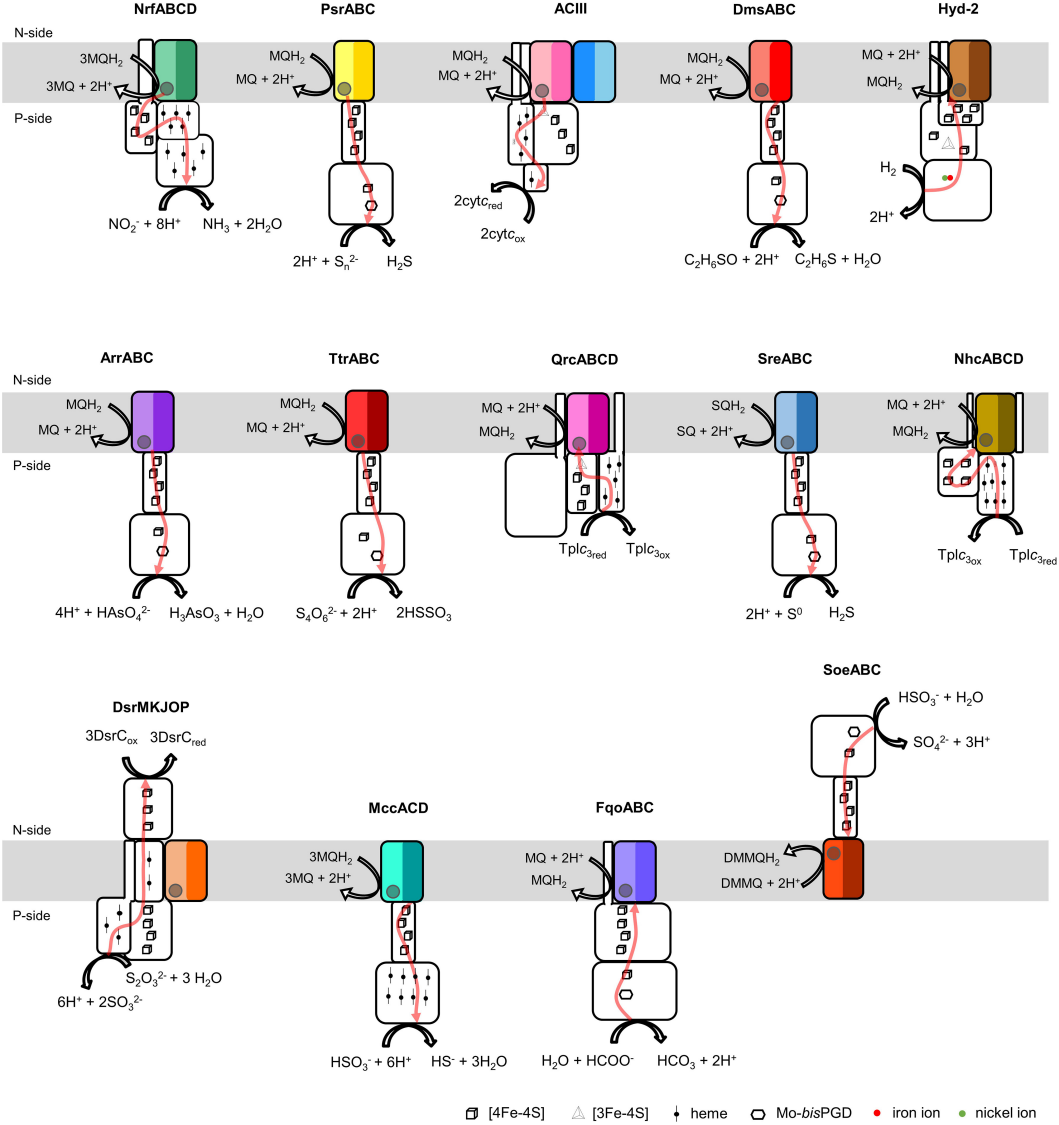
Composition and diversity of NrfD-like subunit-containing complexes. Schematic representation of periplasmic nitrite reductase (NrfABCD), polysulfide reductase (PsrABC), respiratory alternative complex III (ACIII), DMSO reductase (DmsABC), group-2 [Ni-Fe] hydrogenase (Hyd-2), arsenate reductase (ArrABC), tetrathionate reductase (TtrABC), quinone reductase complex (QrcABCD), sulfur reductase complex (SreABC), nine-heme cytochrome complex (NhcABCD), dissimilatory sulfite-reductase complex (DsrMKJOP), multiheme cytochrome *c* sulfite reductase (MccACD), formate:quinone oxidoreductase (FqoABC), sulfite dehydrogenase (SoeABC). P-side, positive-side of the membrane; N-side, negative-side of the membrane. The NrfD-like subunits (NrfD, PsrC, ActC, ActF, DmsC, HybB, ArrC, ttrC, QrcD, SreC, NhcC, DsrP, MccD, fqoC, and SoeC) are colored, the two-color tones indicate the two structural repeats, each composed of a four-helix bundle (TMHs 1–4 and TMHs 5–8) and related by a 180° rotation around an axis perpendicular to the membrane. The circle, inside the colored subunits, indicates the presence of a quinone/quinol-binding site. The red arrows represent the pathway for electron transfer. The color code is used in all the following figures and in Tables 1, 2.

**Figure 2:**
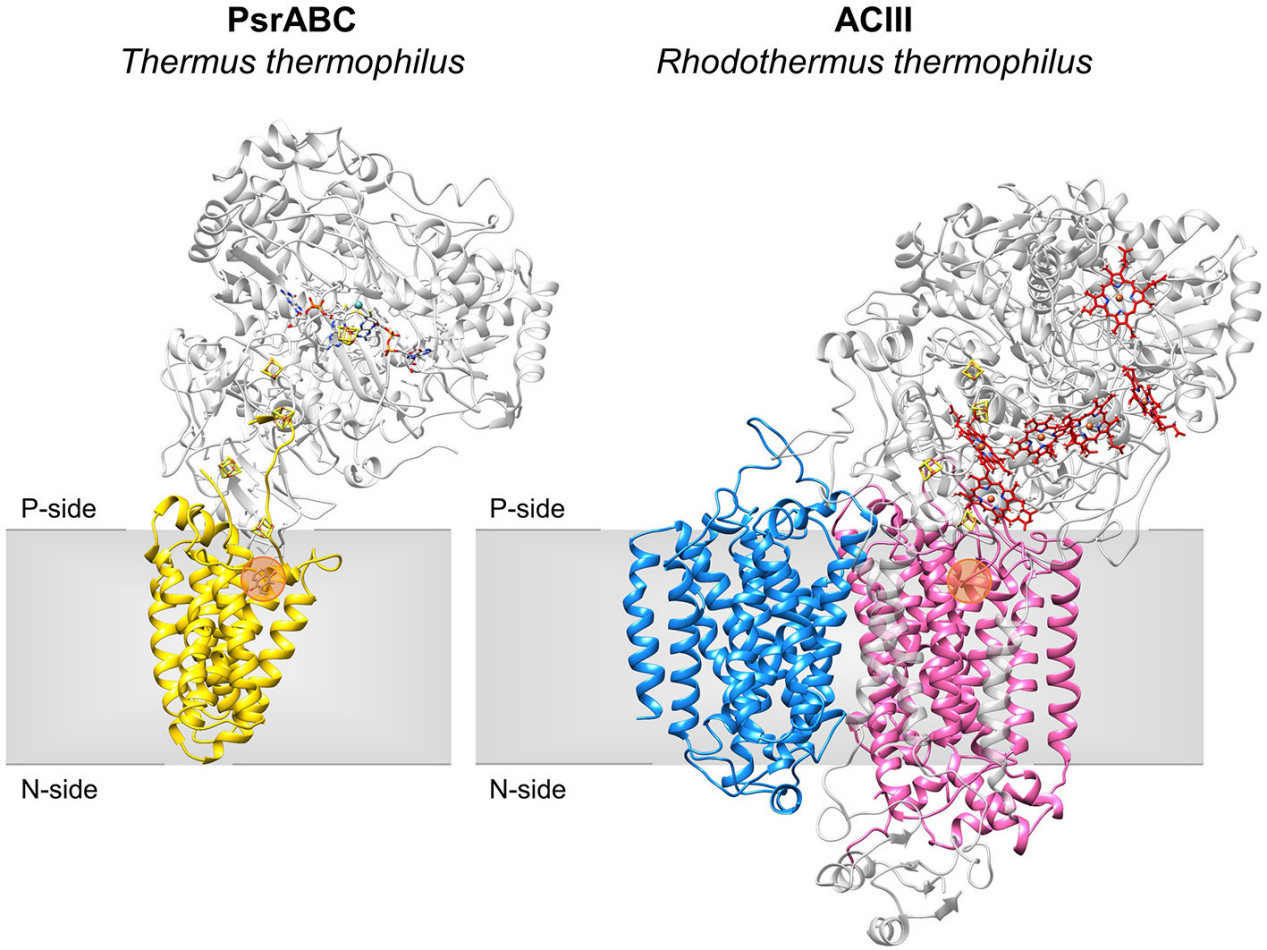
Structures of NrfD-like subunit-containing complexes. Overall structure of PsrABC from *Thermus thermophilus* (left, PDB: 2VPZ) and Alternative Complex III from *Rhodothermus marinus* (right, PDB: 6FOK, ActH was not included for clarity). PsrC (yellow), ActC (pink) and ActF (blue) are NrfD-like subunits. The two complexes are oriented in relation to each other by the superimposition of the PsrC and ActC subunits. Iron-sulfur clusters represented by orange/yellow spheres; Cytochrome *c*-type hemes represented by red sticks; Mo-*bis*PGD cofactor represented by blue/red sticks with molybdenum shown as a blue sphere. Orange dots stand for the quinone-binding site. P-side, positive-side of the membrane; N-side, negative-side of the membrane.

**Figure 3:**
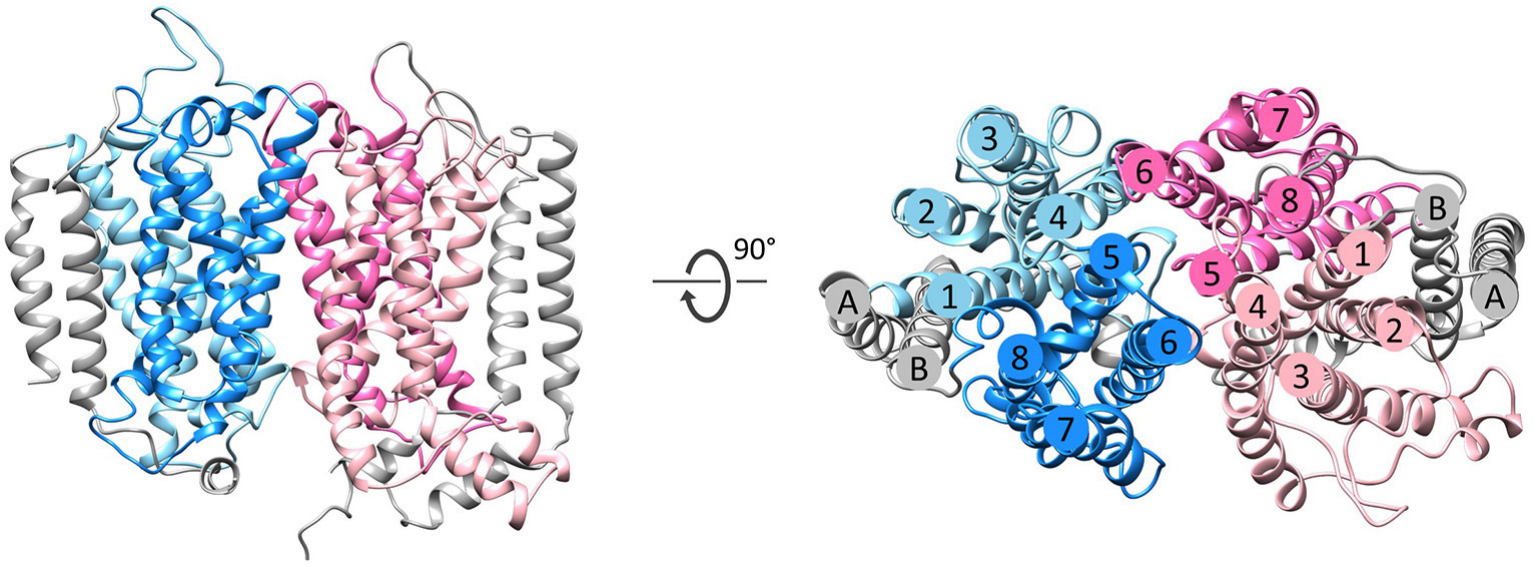
Fold of homologous membrane subunits ActC and ActF. ActC (pink) and ActF (blue) seen from the membrane (left) and from the top (right). The N- and C-terminal helices (TMHs A-B), which form a helix dimer, are colored in gray. The four-helix bundles are shown in light pink/blue (TMHs 1–4) and dark pink/blue (TMHs 5–8).

The NrfD-like subunits are present in many and diverse membrane complexes, widespread in Bacteria and Archaea, that can take part in oxygen, nitrogen, sulfur, arsenate or hydrogen metabolism (Figure 1, Table 1) (Rothery et al., 2008; Refojo et al., 2010, 2019; Marreiros et al., 2016). These subunits thus compose the NrfD family, which was named after the characterization of the periplasmic nitrite reductase (NrfABCD) complex, the first complex recognized to have a NrfD-like subunit (Simon, 2002; Rothery et al., 2008) (Figure 1, NrfABCD). Structures of representatives of the NrfD family were first obtained for the PsrC subunit from the *Thermus thermophilus* polysulfide reductase (PsrABC) complex (Figures 1, 2, PsrABC) and later for the ActC and ActF subunits from the respiratory alternative complex III (ACIII) from *Rhodothermus marinus* (Figure 2, ACIII) and *Flavobacterium johnsoniae* (Sousa et al., 2018; Sun et al., 2018) and for the photosynthetic ACIII from *Roseiflexus castenholzii* (Shi et al., 2020) (Figure 1, ACIII). The structural data showed these membrane subunits have 8 common transmembrane helixes, organized in two four-helix bundles (TMHs 1–4 and TMHs 5–8) related by a 180° rotation around an axis perpendicular to the membrane (Sousa et al., 2018) (Figure 3), and contain one quinone/quinol-binding site close to the periphery of the membrane at the side at which the peripheral subunits are bound to. ActF does not contain any quinone/quinol binding site and it is the only NrfD-like subunit present in a complex in which another subunit of this type (which contains a quinone/quinol-binding site) is present. In addition, for all these subunits, putative ion-conducting pathways were proposed (Calisto et al., 2021). NrfD-like proteins also function as the link of the peripheral subunits to the membrane (Figure 1).

**Table 1:** NrfD-like subunit-containing complexes.

**Color code**		**Complex**	**Activity**	**NrfD-like subunit**	**Catalytic subunit**	**Iron-sulfur subunit**	**Other subunit**
		**Alternative complex III**	Quinol:cytochrome *c*/HiPIP oxidoreductase	**ActC**	**ActA** (5x heme c)	**ActB** (1x [3Fe-4S]^1+/0^, 3x [4Fe-4S]^2+/1+^)	**ActD ActE** (1x heme c)
				**ActF**			
		**Polysulfide reductase**	Quinol:polysulfide oxidoreductase	**PsrC**	**PsrA** (1x Mo-*bis*PGD, 1x [4Fe-4S]^2+/1+^)	**PsrB** (4x [4Fe-4S]^2+/1+^)	x
		**Group-2 [NiFe] hydrogenase**	Hydrogen:quinone oxidoreductase	**HybB**	**HybC** ([Ni-Fe])	**HybO** (1x [3Fe-4S]^1+/0^, 2x [4Fe-4S]^2+/1+^) **HybA** (4x [4Fe-4S]2+/1+)	x
		**DMSO reductase**	QUINOL:DMSO OXIDOREDUCTASE	**DmsC**	**DmsA** (1x Mo-*bis*PGD, 1x [4Fe-4S]^2+/1+^)	**DmsB** (4x [4Fe-4S]^2+/1+^)	x
		**Arsenate reductase**	QUINOL:ARSENATE OXIDOREDUCTASE	**ArrC**	**ArrA** (1x Mo-*bis*PGD, 1x [4Fe-4S]^2+/1+^)	**ArrB** (4x [4Fe-4S]^2+/1+^)	x
		**Dissimilatory sulfite-reductase complex**	thiosulfate:DsrC oxidoreductase	**DsrP**	**DsrJ** (3x heme c) **DsrK** (1x [4Fe-4S]^3+/2+^, 3x [4Fe-4S]^2+/1+^)	**DsrO** (4x [4Fe-4S]2+/1+)	**DsrM** (2x heme *b*)
		**Quinone reductase complex**	TpIc_3_:quinone oxidoreductase	**QrcD**	**QrcA** (6x heme *c*)	**QrcC** 1x [3Fe-4S]^1+/0^, 3x [4Fe-4S]^2+/1+^)	**QrcB**
		**Sulfur reductase complex**	Quinol:sulfur oxidoreductase	**SreC**	**SreA** (1x Mo-*bis*PGD, 1x [4Fe-4S]^2+/1+^)	**SreB** (4x [4Fe-4S]^2+/1+^)	x
		**Periplasmic nitrite reductase**	Quinol:nitrite oxidoreductase	**NrfD**	**NrfA** (5x heme *c*)	**NrfC** (4x [4Fe-4S]^2+/1+^)	**NrfB** (5x heme *c*)
		**Tetrathionate reductase**	Quinol:tetrathionate oxidoreductase	**TtrC**	**TtrA (**1x Mo-*bis*PGD, 1x [4Fe-4S]^2+/1+^)	**TtrB** (4x [4Fe-4S]^2+/1+^)	x
		**Nine-heme cytochrome complex**	Tpi*c*_3_:quinone oxidoreductase	**NhcC**	**NhcA** (9x heme *c*)	**NhcB** (4x [4Fe-4S]^2+/1+^)	**NhcD**
		**Sulfite dehydrogenase**	Sulfite:quinone oxidoreductase	**SoeC**	**SoeA** (1x Mo-*bis*PGD, 1x [4Fe-4S]^2+/1+^)	**SoeB** (4x [4Fe-4S]^2+/1+^)	x
		**Multiheme cytochrome** ***c*** **sulfite reductase**	Quinol:sulfite oxidoreductase	**MccD**	**MccA** (8x heme *c*)	**MccB** (4x [4Fe-4S]^2+/1+^)	x

Besides *Escherichia coli* NrfABCD (Hussain et al., 1994; Clarke et al., 2007), ACIII and PsrABC complexes, the enzymes containing a NrfD-like subunits are dimethyl sulfoxide (DMSO) reductase (DmsABC), tetrathionate reductase (TtrABC), sulfur reductase complex (SreABC), sulfite dehydrogenase (SoeABC) and arsenate reductase (ArrABC) complexes, members of the so called complex iron-sulfur molybdoenzyme (CISM) family, which have a similar subunit composition to that of PsrABC complex (Figure 1) (Rothery and Weiner, 1993; Weiner et al., 1993; Berks, 1996; Hensel et al., 1999; Hinsley and Berks, 2002; Laska et al., 2003; Geijer and Weiner, 2004; Guiral et al., 2005; Duval et al., 2008; Dahl et al., 2013; Grimaldi et al., 2013; Tang et al., 2013; Steinmetz et al., 2014; Kurth et al., 2015; Boughanemi et al., 2020). The subunits and prosthetic groups of these enzymes are specified in Table 1. DmsABC is a quinol:DMSO oxidoreductase with its peripheral DmsAB subunits facing the P-side of the membrane (Rothery and Weiner, 1993; Berks, 1996). TtrABC, expressed under anaerobic conditions in Proteobacteria (Marreiros et al., 2016), catalyzes the endergonic reduction of tetrathionate to thiosulfate with the concomitant oxidation of quinol to quinone. As DmsAB, TtrAB subunits are facing the P-side of the membrane (Hensel et al., 1999; Hinsley and Berks, 2002). SreABC, characterized in *Acidianus ambivalens*, was proposed to have quinol:sulfur oxidoreductase activity and SreAB were suggested to be located at the P-side of the membrane (Laska et al., 2003). SoeABC is a sulfite:quinone oxidoreductase complex described in *Aquifex aeolicus* and *Allochromatium vinosum* (Dahl et al., 2013; Boughanemi et al., 2020). SoeAB subunits were hypothesized to be located at the negative-side (N-side) of the membrane. ArrABC, involved in arsenate respiration, seems to have the peripheral ArrAB subunits at the P-side of the membrane (Stolz et al., 2006; Duval et al., 2008).

**Table 2:** Amino acid residues conservation in NrfD-like subunit-containing complexes.

**Color code**		**Complex**	**NrfD-like subunit**	**KEGG Template**	**Quinone-binding site (TMHs 1-4)**	**N-side ion-translocation half-channel (TMHs 1-4)**	**P-side ion-translocation half-channel (TMHs 5-8)**	**Residues conservation**	*Amino acid residues variations	**# TMH**	**# sequences**
		**Alternative Complex III**	**ActC**	rmr:rmar_0223	His139, Ser164, Asp169, Asp253	His97, Thr100, Ser103, Arg114, Arg119, Glu122, Thr125, Tyr176, Ser180, Asp191, Arg196, Asp197, His247	Tyr270, Tyr323, Glu326, Glu338, Glu394, Arg395	>95%	x	10	521
			**ActF**	rmr:rmar_0226	x	Ser80, His88, Arg100, Glu103*, Tyr165, Thr170, Ser180*, Asp184*, Ser199, Tyr206	Ser227, Thr228, Tyr233*, Ser238, Tyr284, Tyr290, Glu300, Glu301, Arg308, His355*, Asp358*	>80%	*E103-73%, R/Y-7% *S180-71.9%, R/T/E-10% *D184-77%, E/K/S/Y-5% *Y233-76%, D/T/S-4% *H355-69%, R/T/E/S/K-14%*D358-75%, E/H/R-20%	10	516
		**Polysulfide reductase**	**PsrC T**	tth:tt_c0153	Asn18*, His21*, Glu67, Ser87, Tyr130	Arg48*, Thr50, Asp60, Thr102*, Tyr107, Arg114*, Ser124	Thr155, Ser183, Tyr190, Glu197*, Glu205*, Tyr210, Arg239	>80%, model sequence	*N18-73%, S-13% *H21-40%, Y-60% *R48-33%, S-53% *T102-13%, S-73% *R114-53%, K-40% *E197-33%, S/T-60% *E205-33%, T/S/R-67%	8	15
			**PsrC W**	wsu:ws0118	Tyr23, Asp76, Ser94, Tyr159, Thr160	Ser30, Lys41*, Asp52, Lys56, Tyr106, Glu146	Ser185, Ser188, Glu225, Tyr307, Tyr310, Arg305	>95%, model sequence	*K41-88%, E/R-12%	8	33
		**Group-2 [NiFe] hydrogenase**	**HybB**	eco:b2995	Asp58, Ser105, Asp109, Ser129, Glu133, Ser190	Thr63, Tyr77, Tyr84, His85, Arg89, Ser95, Tyr99, Tyr141, Glu148, Glu155, His184, Ser186, Ser187	Arg265, Glu268*, Glu291, Lys198, His200, Arg329	>95%	*E268-86%, D-13%	10	320
		**DMSO reductase**	**DmsC**	eco:b0896	Ser62, His65, Ser82, Glu87	Thr14, Glu39, Arg43, Ser90, Lys109, Thr116*, Ser111, Arg115, Thr122*	Thr135, Thr151, Thr158, Ser195, Ser199, Glu205, Asp222, Arg230, Glu267, Arg271, Tyr275	>80%, model sequence	*T116-15%, K/S/R/E/H-58% *T122-39%, S-19%	8	793
		**Arsenate reductase**	**ArrC**	aeh:mlg_0214	Tyr57, Glu107, Ser126	Ser64, Tyr83, Lys87, Arg88, Tyr135, Lys145, Asp153, Lys160*	Tyr198, Tyr200, Ser206, Arg256, Arg324, Ser279*	>95%	*K160-44%, H/R-44% *S279-56%, T-33%	10	9
		**Dissimilatory sulfite-reductase complex**	**DsrP1**	dvu:dvu1286	Tyr52, Asp106, Ser124, Asp129	Thr57, Tyr74, Glu89, Tyr136, Arg152, Lys162, Ser168*, His176, Thr179	Arg189, Thr194, Arg200, Ser204, Glu254, His266, Lys329	>95%	*S168-93%	10	66
			**DsrP2**	alv:alvin_1262	His57, Asp110, Ser128, Thr178	Ser68, Ser75, Tyr85, Ser92, Tyr137, Tyr147, Arg171, Thr175, Thr176	Ser204, Tyr248, His254, Tyr259, Tyr277*	>95%	*Y277-84%, H-9%	10	36
		**Quinone reductase complex**	**QrcD**	dvu:dvu0692	Asp70, Asp120*, Ser138, Glu142, Ser202	Arg87*, Tyr88, Asp93*, Tyr110, Tyr150, Glu157, Glu164, Arg166, His182, Ser196, His199, Ser196	Ser234, Tyr227, Tyr278, Glu319, Lys282, Asp285, Thr286, Tyr311, Arg358	>95%	*R87-53%, K/Y/S-47% *D93-68%, K/E-32% *D120-91%, E-9% *R166-62%, K-38%	10	35
		**Sulfur reductase complex**	**SreC**	aamb:d1866_ 07880	Tyr55, Ser110, Ser128, Arg129	Thr63, Ser66, Tyr68, Ser70, Glu73, Arg91, Glu93, Tyr137, Glu154, Lys155, Asp206	Glu222, Ser228, Ser330, Ser235, Tyr237, Thr311, Asp380, Tyr389	>80%, model sequence	x	10	21
		**Periplasmic nitrite reductase**	**NrfD**	eco:b4073	Asp15, Tyr21, His74, Ser92, Tyr162, Thr163	Ser28, Arg39*, Tyr104, His118*, Glu149*	Ser188, S191, Glu228, Lys246, Tyr260, Tyr311, Arg306	>80%, model sequence	*R39-65%, K-35% *H118-26%, K/D/E/T/S/R-47% *E149-78%, T/D/S-14% *K246-77%, S/R-16%	8	411
		**Tetrathionate reductase**	**TtrC**	sty:sty1737	Tyr26, Asp75, Ser93, Tyr146, Thr147, Glu150	Glu54, Thr63, Thr106, Lys119, Arg125, Thr129, Ser136	Arg157, Ser162*, Ser172*, Ser220, Arg280, Lys292	>80%, model sequence	*S162-46%, T-41% *S172-66%, T-34%	9	292
		**Nine-heme cytochrome complex**	**NhcC**	dds:ddes_2040	Asp68, Asp119, Ser137, Glu141, Ser200	Tyr87*, Arg99, Thr104, Tyr109, Tyr149, Glu156, Glu163, Thr195, His197	Ser218, Ser228, Tyr278, Lys282, Glu306, Arg345	>80%	*Y87-58%, H-35%	10	81
		**Sulfite dehydrogenase**	**SoeC**	aae:aq_1231	Ser6, His65, Glu87, Thr132, Ser82, Ser85	Tyr25, Lys41, Ser47, Ser51, Thr97, Tyr106, Glu114, Thr116, Thr121, Ser128	Glu144*, Thr150, Arg224, Lys228, Glu239, Arg297, His306	>80%, model sequence	*E144-46%, R/S-29%	8	239
		**Multiheme cytochrome** ***c*** **sulfite reductase**	**MccD**	wsu:ws0382	Tyr24, Asp75, Ser93, Tyr161, Thr162	Ser31, Arg42*, Lys55, Ser58, Ser61, Thr64, Tyr105, Ser109, Ser119, Tyr137, Arg141, Arg144*, Glu148, Thr151, Thr157	Ser187, Ser190, Glu227, Ser237, Lys246, Ser248, Tyr257, Arg306	>95%, model sequence	*R42-84%, K-11% *R144-87%, E/Y/K-13%	8	79
		**Formate:quinone oxidoreductase**	**FqoC**	cte:ct0494	Tyr16, Asp65, Ser81, Tyr147, Thr148	Thr26, Ser30, His34, Glu37*, Asp40, Arg41, Ser45*, Ser50, Tyr93, Tyr100, Glu127, Lys131, Ser138	Ser173, Glu212, Tyr219, Arg231, Glu235, His239, Glu259, Tyr295, Arg293	>80%, model sequence	*E37-13%, T/S/D-88% *S45-10%, R/K-90%	8	114

*Amino acid residues, putatively involved in ion-conducting pathways and quinone/quinol-binding are represented in black (conserved residues in respective sequence alignments), gray (amino acid residues present in the model able to conduct protons, but not conserved in respective sequence alignments) and red (equivalent to Arg395^ActC^)*.

The multiheme cytochrome *c* sulfite reductase complex (MccACD), biochemically characterized from *W. succinogenes* and *Shewanella oneidensis* (Kern et al., 2011; Shirodkar et al., 2011) (Figure 1, MccACD, Table 1), the quinone reductase complex (QrcABCD), homologous to ACIII and present in sulfate-reducing bacteria, are other enzymes with NrfD-like subunits. Qrc catalyzes the transfer of electrons from a type-1 tetraheme cytochrome *c*_3_ (TpI*c*_3_) to quinone (Figure 1, Qrc) and so the peripheral subunits are facing the P-side of the membrane (Pereira et al., 2011; Venceslau et al., 2011). Group-2 [NiFe] hydrogenase (Hyd-2), produced under anaerobic conditions, is a hydrogen:quinone oxidoreductase complex with the peripheral subunits facing the P-side of the membrane (Laurinavichene et al., 2002; Sargent, 2016; Beaton et al., 2018) (Figure 1, Hyd-2, Table 1). The nine-heme cytochrome complex (NhcABCD), a TpI*c*_3_:quinone oxidoreductase, is a member of a family of transmembrane complexes with multiheme cytochrome *c* subunit, described in *Desulfovibrio* species (Matias et al., 1999, 2005; Saraiva et al., 2001; Bento et al., 2003; Pereira et al., 2011) (Figure 1, Nhc, Table 1). The dissimilatory sulfite-reductase complex (DsrMKJOP) is a transmembrane complex with peripheral subunits at both sides of the membrane (Grein et al., 2010). DsrJ and DsrO subunits are anchored to the P-side of the membrane (Pires et al., 2006). DsrM is a di-heme cytochrome *b* membrane subunit (Pires et al., 2006). DsrK is facing the N-side of the membrane (Mander et al., 2002; Pires et al., 2006). DsrK is able to reduce the small protein DsrC, while DsrJ was suggested to interact with thiosulfate (Bamford et al., 2002; Denkmann et al., 2012; Venceslau et al., 2014) (Figure 1, DsrMKJOP, Table 1). Since DsrP and DsrM are membrane subunits with hypothetical quinone-binding sites, a quinone cycling of reduction and oxidation between DsrMK and DsrJOP was proposed to take place (Grein et al., 2013).

In this work we thoroughly analyze the primary structures of the members of the NrfD family and predicted the respective tertiary structures, the data provided allowed a deep and broad discussion on the presence of ion-conducting pathways and quinone/quinol-binding sites, which impacts on the function of the several complexes, namely in their ability to transduce energy.

## Results and Discussion

### Taxonomic Distribution

**Figure 4:**
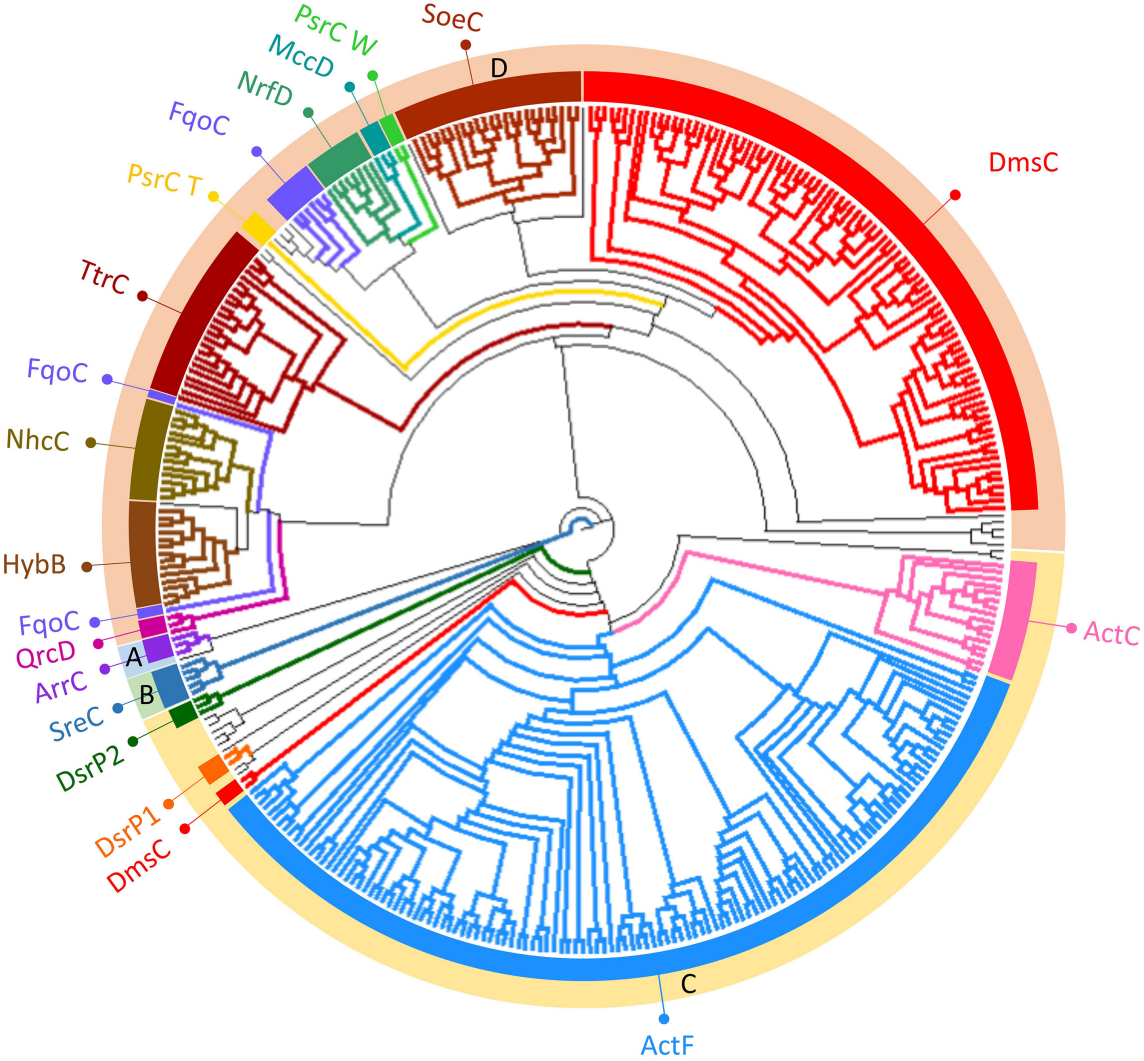
Neighbor-Joining dendrogram of the NrfD-like subunits. The dendrogram was obtained using 551 representative sequences of NrfD-like subunit sequences. Branches are colored according to the color code indicated in the Figure 1. The external ring of the dendrogram indicates the four major identified groups discussed in the text (groups A to D).

NrfD-like subunits are the anchor protein of several modular complexes, which take part in a vast array of metabolisms, such as the oxygen, nitrogen, sulfur, arsenate and hydrogen cycles (Rothery et al., 2008; Refojo et al., 2010, 2019; Marreiros et al., 2016) (Figure 1). In this work, we performed sequence alignments and taxonomic profiling to investigate the distribution of the NrfD-like subunit-containing complexes in microbial species. We gathered 4,545 NrfD-like amino acid sequences present in the genomes of 1,822 distinct species (96% Bacteria, 4% Archaea), with an average of 2.5 NrfD-like subunits per organism. In order to reduce the size of the obtained dataset, the 4,545 amino acid sequences were clustered according to their identity (50% identity) and 551 representative sequences were aligned, allowing to generate the correspondent Neighbor-Joining (NJ) dendrogram. From the obtained dendrogram we were able to identify several branches belonging to different groups of NrfD-like subunit-containing complexes (Figure 4).

**Figure 5:**
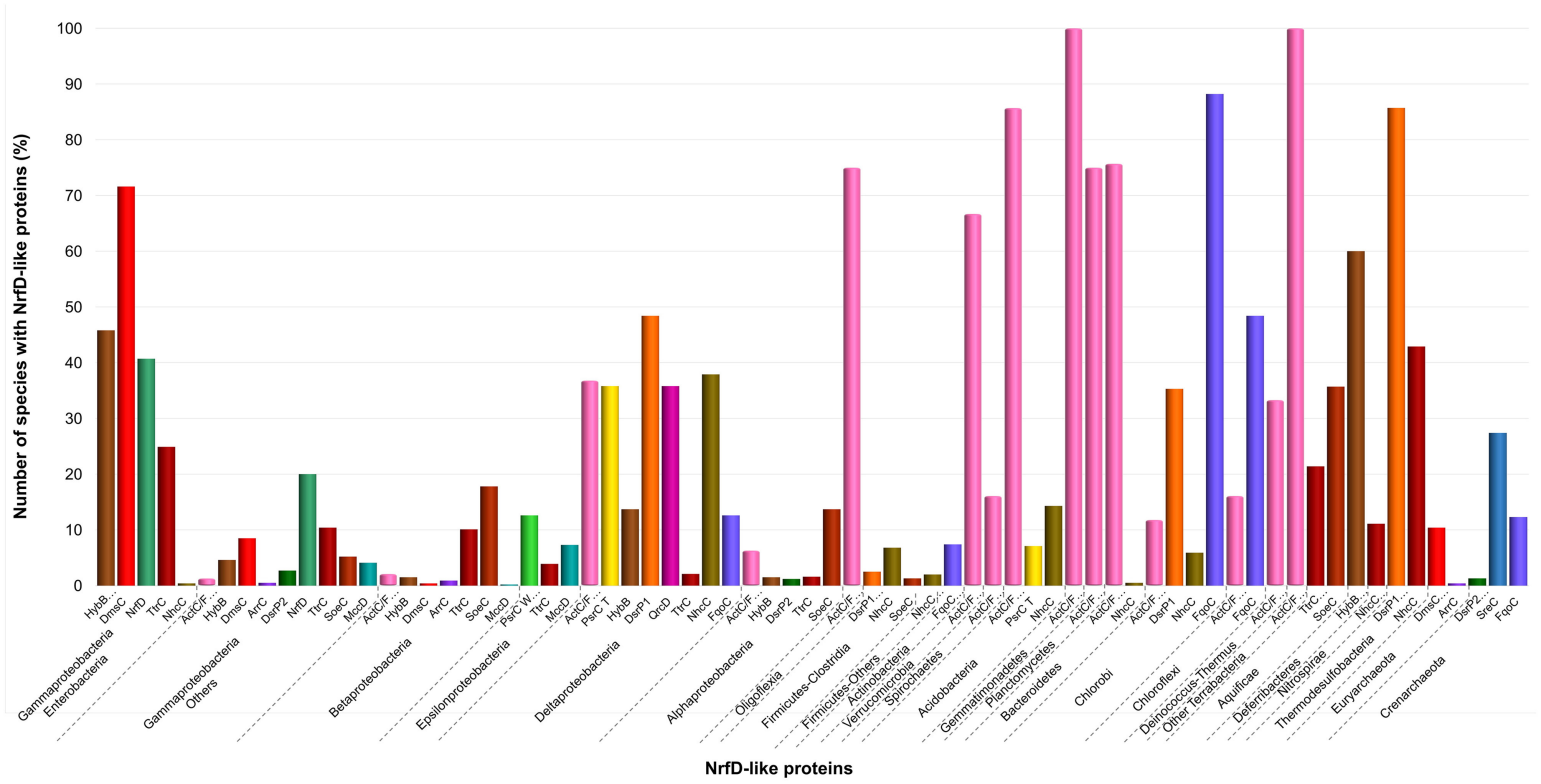
Distribution of NrfD-like subunits in Prokaryotes. The diagram indicates the percentage of the species within each phylum containing at least one gene encoding a NrfD-like subunit. The color code is indicated in the figure.

We observed that close to the root of the dendrogram, the sequences of NrfD-like subunits separated into four major groups (Figure 4, dendrogram groups A–D). The amino acid sequences of SreC (21 sequences) (Figure 4, group A) and ArrC (9 sequences) (Figure 4, group B) seem to be less related with the others NrfD-like subunits and constitute two distinct groups. The SreABC complex was biochemically characterized from the sulfur-dependent archaeon *A. ambivalens* (Laska et al., 2003) and genes coding for SreC subunit were only identified in archaea, in 21 species (27% of Crenarchaeota species) (Figure 5, SreC). ArrAB complex was described as a periplasmic complex, which is associated with the transmembrane ArrC subunit only in few microorganisms (Duval et al., 2008). In agreement, we identified nine genes coding for ArrC subunit, distributed in Gammaproteobacteria (four sequences), Betaproteobacteria (four sequences) and Euryarchaeota (one sequence) species (Figure 5, ArrC).

The third group (Figure 4, group C) englobes the amino acid sequences of ActC (521 sequences), ActF (521 sequences), DsrP (88 sequences) and DmsC (42 sequences) subunits. Intriguingly, we identified two branches of DsrP amino acid sequences, which we called DsrP1 and DsrP2 (Figure 4). The amino acid sequence of DsrP subunit, biochemically characterized from *Desulfovibrio vulgaris* DsrMKJOP complex, was found in the DsrP1 branch (66 amino acid sequences of sulfate-reducing bacteria). Besides their presence in Deltaproteobacteria (46 species, 48%), genes coding for DsrP1 subunit were identified in species from Thermodesulfobacteria (6 species, 86%), Chlorobi (6 species, 35%) and Firmicutes-Clostridia (6 species, 3%) phyla (Figure 5, DsrP1). Genes coding for DsrP2 subunit were observed in 36 species of sulfur-oxidizing bacteria and from Archaeoglobus genus (23 species, 3% of Gammaproteobacteria; 10 species, 1% of Alphaproteobacteria; 3 species of 1% of Euryarchaeota species) (Figure 5, DsrP2). The amino acid sequences of ActC and ActF subunits (521 sequences) were naturally separated into two branches (Figure 4, ActC and ActF). Genes coding for ActC and ActF subunits were identified in species from Gammaproteobacteria-Others (11 species, 1%), Betaproteobacteria (10 species, 2%), Deltaproteobacteria (35 species, 37%), Alphaproteobacteria (46 species, 6%), Oligoflexia (nine species, 75%), Verrucomicrobia (10 species, 67%), Spirochaetes (14 species, 16%), Acidobacteria (12 species, 86%), Gemmatimonadetes (three species, 100%), Planctomycetes (24 species, 75%), Bacteroidetes (290 species, 76%), Chlorobi (two species, 12%), Chloroflexi (six species, 16%), Deinococcus-Thermus (11 species, 33%) and Terrabacteria (three species, 100%) (Figure 5, ActC/F). Group C of the dendrogram also includes 42 amino acid sequences identified as DmsC subunit (Figure 4, group DmsC), namely the DmsC amino acid sequence from *Halobacterium* sp. strain NRC-1 (Müller and DasSarma, 2005). Genes coding for DmsC present in group C are all observed in 24 archaeal species from Euryarchaeota (10%) (Figure 5, DmsC).

The fourth group (Figure 4, group D) is composed of amino acid sequences of PsrC (48 sequences), DmsC (793 sequences), NrfD (411 sequences), TtrC (292 sequences), NhcC (81 sequences), HybB (320 sequences), QrcD (35 sequences), SoeC (239 sequences) and MccD (79 sequences) subunits. As observed before (Duarte et al., 2018), the amino acid sequences of PsrC subunit forms two separate branches, one represented by PsrC from *W. succinogenes* and the other pictured by PsrC from *T. thermophilus*. The amino acid sequence of PsrC subunit from *T. thermophilus* PsrABC complex, clusters within branch PsrC T with other 34 amino acid sequences (Figure 4, PsrC T) and PsrC from *W. succinogenes* PsrABC complex is present in branch PsrC W (Figure 4, PsrC W; 33 sequences). Genes coding for PsrC T subunits were found in 34 species (36%) from Deltaproteobacteria and one species (7%) from Acidobacteria, while genes coding for PsrC W subunits were observed in 26 species (13%) from Epsilonproteobacteria (Figure 5, PsrC T and PsrC W).

Genes coding for bacterial DmsC and NrfD subunits were identified in 80% (444 species) and 61% (383 species) of species from Gammaproteobacteria, respectively (Figure 5, DmsC and NrfD). Amino acid sequences of TtrC subunits were expected to be produced in 291 species, distributed in Proteobacteria (219 species, 35% Gammaproteobacteria; 47 species, 10% Betaproteobacteria; eight species, 4% Epsilonproteobacteria; two species, 2% Deltaproteobacteria; 12 species, 2% Alphaproteobacteria), Aquificae (three species, 21%) and Armatimonadetes (one species, 50%) phyla (Figure 5, TtrC). NhcC subunit coding genes were found in 64 bacteria species (36 from Deltaproteobacteria, 38%; 16 from Firmicutes-Clostridia, 9%; two from Acidobacteria, 14%; 2 from Bacteroidetes; one from Chlorobi, 6%; 1 from Nitrospirae, 11%; three from Thermodesulfobacteria, 43%) (Figure 5, NhcC). Genes coding for HybB subunit were observed in 314 species, 277 from Gammaproteobacteria (50%), 7 from Betaproteobacteria (2%), 13 from Deltaproteobacteria (4%), 11 from Alphaproteobacteria (2%), 2 from Acidobacteria (14%), 1 from Aquificae (7%) and three from Deferribacteres (60%) (Figure 5, HybB). The presence of genes coding for QrcD subunit was restricted to 35 species (36%) from Deltaproteobacteria (Figure 5, QrcD). Genes coding for SoeC subunit were present in 236 species from Proteobacteria (45 species from Gammaproteobacteria, 5%; 83 species from Betaproteobacteria, 18%; 100 species from Alphaproteobacteria, 14%), Firmicutes-Clostridia (three species, 1%) and Aquificae (five species, 36%) phyla (Figure 5, SoeC). While genes coding for MccD subunit were identified in 51 species (35 from Gammaproteobacteria, 4%; 15 from Epsilonproteobacteria, 7%) (Figure 5, MccD).

In group D of the dendrogram (Figure 4, FqoC), we were able to identify 110 amino acid sequences present in an uncharacterized complex, that we tentatively assigned as formate:quinone oxidoreductase complex (Figure 1, FqoABC). Our assignment is based on the observations that the complex is possibly composed of three subunits: two peripheral FqoAB subunits homologous to FdnGH subunits from *E. coli* formate dehydrogenase complex (Fdn-N) and a FqoC subunit, a NrfD-like subunit with eight predicted TMHs. Genes coding for FqoC were identified in 110 species present in three different dendrogram groups: FqoC1 includes genes coding for FqoC subunit from Actinobacteria (59 species, 7%), Chlorobi (15 species, 88%) and Crenarchaeota (9 species, 12%) phyla; FqoC2 gathers the genes coding for FqoC from Chloroflexi phylum (15 species, 48%); and FqoC3 includes genes coding for FqoC subunit from Deltaproteobacteria phylum (12 species, 13%) (Figure 5, FqoC).

### Homology Models

The NrfD-like subunits are transmembrane proteins, with 8 to 10 TMHs, that interact with quinones but do not have redox cofactors. The structures of *T. thermophilus* PsrC and *R. marinus* ActC and ActF revealed details of the overall architecture of these NrfD-like subunits: the common 8 TMHs are organized in two four-helix bundles (TMHs 1–4 and TMHs 5–8), which form two structural repeats related by a 180° rotation around an axis perpendicular to the membrane layer plane (Jormakka et al., 2008; Sousa et al., 2018). Two additional TMHs (TMHs A-B), present in ActC and ActF, cross each other at an angle of ~45° at the periphery of each subunit (Sousa et al., 2018) (Figure 3). ActC and ActF subunits have their N- and C-terminal located at N-side of the membrane, while PsrC have both termini located at the P-side of the membrane. *T. thermophilus* PsrC and *R. marinus* ActC subunits contain one quinol-binding site at the P-side of the membrane of the first four-helix bundle (Jormakka et al., 2008; Sousa et al., 2018) (Figure 2).

We performed structural homology models of the NrfD-like subunits from *W. succinogenes* PsrABC, *E. coli* NrfABCD, *E. coli* DmsABC, *E. coli* Hyd-2, *Salmonella enterica* TtrABC, *A. ambivalens* SreABC, *A. aeolicus* SoeABC, *D. vulgaris* QrcABCD, *D. vulgaris* DsrMKJOP1, *A. vinosum* DsrMKJOP2, *Desulfovibrio desulfuricans* NhcABCD, *Alkalilimnicola ehrlichii* ArrABC, *W. succinogenes* MccACD and *Chlorobaculum tepidum* FqoABC complexes. The homology models were calculated in Phyre2 without imposing any template. In all cases the randomly selected template was the structure from *R. marinus* ActC subunit. The homology models have confidence scores higher than 90%. The final models presented the common 8 TMHs organized in a similar arrangement as those of ActC, ActF and PsrC subunits. As in the case of *T. thermophilus* PsrC subunit, we predicted only 8 TMHs for *W. succinogenes* PsrC, *E. coli* DmsC, *E. coli* NrfD, *A. aeolicus* SoeC, *W. succinogenes* MccD and *C. tepidum* FqoC subunits. *S. enterica* TtrC subunit was predicted to have 9 TMHs, with the N-terminal at the P-side and the C-terminal at the N-side of the membrane, the extra TMH is equivalent to TMH B from ActC. The other NrfD-like subunits (*E. coli* HybB, *A. ehrlichii* ArrC, *D. vulgaris* DsrP1, *A. vinosum* DsrP2, *D. vulgaris* QrcD, *A. ambivalens* SreC and *D. desulfuricans* NhcC) have 10 predicted TMHs, the two extra TMHs are equivalent to TMH A and B of ActC and ActF subunits. The models predict for all NrfD-like subunits the presence of the structural repeats, composed of the two four-helix bundles, harboring putative ion-conducting pathways and the existence of a quinone/quinol-binding site (Figure 1, Table 2).

### Quinone-Binding Site

**Figure 6:**
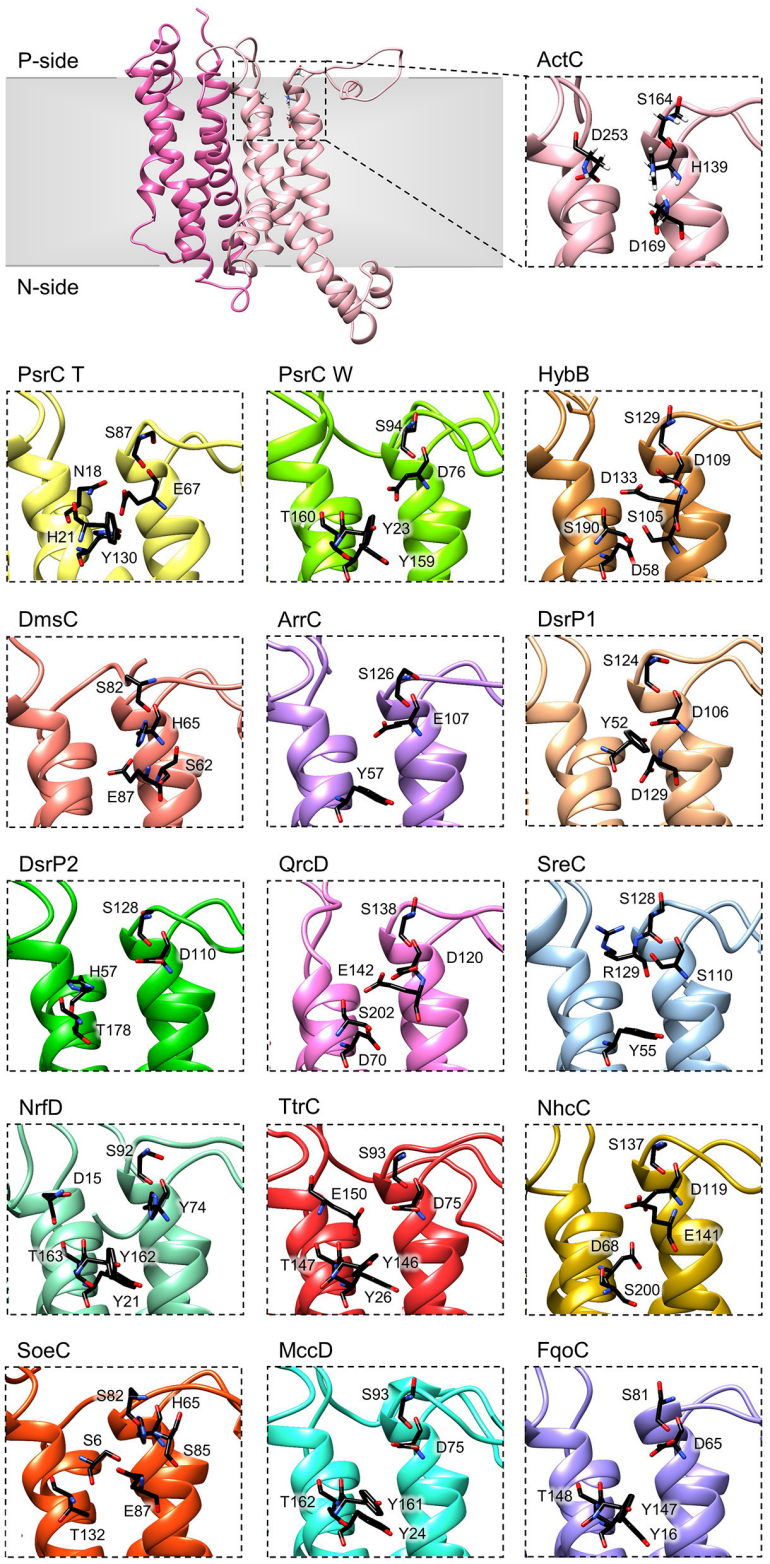
Quinone-binding site. Zoomed views of structural models of NrfD-like subunits showing the respective putative quinone/quinol-binding sites, located close to the positive-side (P-side) of the membrane. The SoeC subunit is expected to be in an inverted orientation toward the membrane in relation to the other NrfD-like subunits and thus its quinone/quinol binding site is located close to the negative-side (N-side). The amino acid residues composing the different quinone/quinol-binding sites are indicated and depicted as sticks. Please note that all models are oriented with the P-side of the membrane at the top of each panel, except for SoeC that is oriented with the N-side of the membrane at the top of the respective panel.

The crystal structures of *T. thermophilus* PsrC co-crystallized with either menaquinone-7 or ubiquinone-1 showed that the quinone-binding site is located in the first four-helix bundle of PsrC (TMHs 1-4) on the P-side of the membrane, in close proximity to the [4Fe-4S]^2+/1+^ cluster of PsrB subunit (Jormakka et al., 2008). Highly conserved amino acid charged residues, present in this region, were suggested as essential for quinone-binding and coordination: His21^PsrCT^, Asn18^PsrCT^ and Tyr130^PsrCT^ in *T. thermophilus* PsrC and His139^ActC^, Asp169^ActC^ and Asp253^ActC^ in *R. marinus* ActC (Jormakka et al., 2008; Sousa et al., 2018) (Figure 6, ActC and PsrC T).

To further investigate structurally relevant elements and/or amino acid residues in the quinone-binding site, we analyzed the obtained structural homology models of the NrfD-like subunits and performed sequence alignment to identify the conserved residues involved in the binding and coordination of quinone/quinol molecule.

We identified for all NrfD-like subunits, with the exception of ActF subunit, the only NrfD-like that has been shown not to interact with quinones/quinols and part of a complex in which another NrfD-like subunit is present, the presence of quinone/quinol-binding site in the same spatial position of those observed for *T. thermophilus* PsrC and *R. marinus* ActC. These are all located in the first four-helix bundle (TMHs 1–4), close to the P-side of the membrane. In the case of SoeC the quinone/quinol-binding site is also present in the first four-helix bundle (TMHs 1–4) at the same special position of sites present in the other NrfD-like subunits, however it is expecting to be facing the N-site of membrane. This is because the catalytic subunit of SoeABC complex is predicted to be oriented toward the N-side of the membrane (Dahl et al., 2013; Boughanemi et al., 2020) (Figure 1) and thus SoeC would be expected to have an inverted orientation inside the membrane plane comparing to the other NrfD-like proteins.

We observed, within the predicted quinone/quinol-binding site of all NrfD-like subunits analyzed, the presence of 5, on average, amino acid residues (histidine, arginine, aspartate, glutamate, serine, tyrosine, threonine and asparagine) that may be involved in quinone/quinol-binding and stabilization in each protein (Figure 6, Table 2).

Although histidine, serine, glutamate, aspartate and tyrosine residues were described as being involved in the coordination of quinones in several quinone-interacting complexes, there seems to be no common pattern for quinone-binding (Abramson et al., 2000; Fisher and Rich, 2000; Zhang et al., 2002; Iwaki et al., 2003; Horsefield et al., 2006; Kleinschroth et al., 2008). Nevertheless, we noticed a conserved serine residue in all NrfD-like subunits, close to the entry of the quinone/quinol pocket (Ser164^ActC^, Ser87^PsrC_T^, Ser94^PsrC_W^, Ser129^HybB^, Ser82^DmsC^, Ser126^ArrC^, Ser124^DsrP1^, Ser128^DsrP2^, Ser138^QrcD^, Ser128^SreC^, Ser92^NrfD^, Ser93^TtrC^, Ser137^NhcC^, Ser82^SoeC^, Ser93^MccD^ and Ser81^FqoC^). We hypothesized that this serine residue is involved in quinone coordination, since it is only absent in *R. marinus* ActF subunit, the only NrfD-like that has been shown not to interact with quinone (Sousa et al., 2018). Mutation of Ser94^PsrC_W^ residue in *W. succinogenes* PsrC caused partially inhibition of polysulfide respiration (Dietrich and Klimmek, 2002). We also identified in TMH 2, in the same spatial position in all NrfD-like subunits (again with the exception of ActF), the presence of a glutamate/aspartate/histidine/serine residue (Glu67^PsrC_T^, Glu107^ArrC^, Asp76^PsrC_W^, Asp109^HybB^, Asp106^DsrP1^, Asp110^DsrP2^, Asp120^QrcD^, Asp75^TtrC^, Asp119^NhcC^, Asp75^MccD^, Asp65^FqoC^, His139^ActC^, His65^DmsC^, His65^SoeC^, His74^NrfD^ and Ser110^SreC^) that seems to be required for protonation/deprotonation of the quinone/quinol molecule (Table 2). Mutational studies support the relevance of this position. Replacement of Asp76^PsrC_W^ and His65^DmsC^ residues, in *W. succinogenes* PsrC and *E. coli* DmsC, respectively, blocked quinol oxidation (Rothery and Weiner, 1996; Zhao and Weiner, 1998; Geijer and Weiner, 2004) and lead to full inhibition of polysulfide respiration (Dietrich and Klimmek, 2002). In *T. thermophilus* PsrC structure, Tyr130^PsrC_T^ and His21^PsrC_T^ residues were proposed to act as ligands to menaquinone and pentachlorophenol, a quinone inhibitor (Jormakka et al., 2008), and, in fact, mutation of Tyr23^PsrC_W^ residue, the correspondent residue in *W. succinogenes* PsrC, also inhibit polysulfide respiration (Dietrich and Klimmek, 2002). Additional mutational studies, conducted in *E. coli* DmsABC (Rothery and Weiner, 1996; Zhao and Weiner, 1998), *W. succinogenes* PsrABC (Dietrich and Klimmek, 2002) and *E. coli* Hyd-2 (Lubek et al., 2019), also showed the importance of Glu87^DmsC^, Tyr159^PsrC_W^, Thr160^PsrC_W^ and Asp58^HybB^ residues for quinone/quinol coordination (Rothery and Weiner, 1996; Zhao and Weiner, 1998; Dietrich and Klimmek, 2002; Geijer and Weiner, 2004; Lubek et al., 2019).

Our models indicate that all NrfD-like proteins (except for ActF) are able to interact with quinones/quinols (Figures 1, 6).

### Ion Translocation Pathways

NrfD-like subunit-containing complexes were hypothesized to be capable of ion-translocation across the membrane (Calisto et al., 2021) and, in fact, proton-conducting pathways have been identified in the structures of the PsrC, ActC and ActF subunits (Jormakka et al., 2008; Sousa et al., 2018; Sun et al., 2018; Shi et al., 2020). Proton-conducting pathways are formed by amino acid residues with side chains that can establish hydrogen bonds, constituting a hydrogen bond network. This allows proton transfer by a Grotthuss-type mechanism, which involves successive breaking and concomitant formation of hydrogen bonds (de Grotthuss, 1806; Cukierman, 2006).

**Figure 7:**
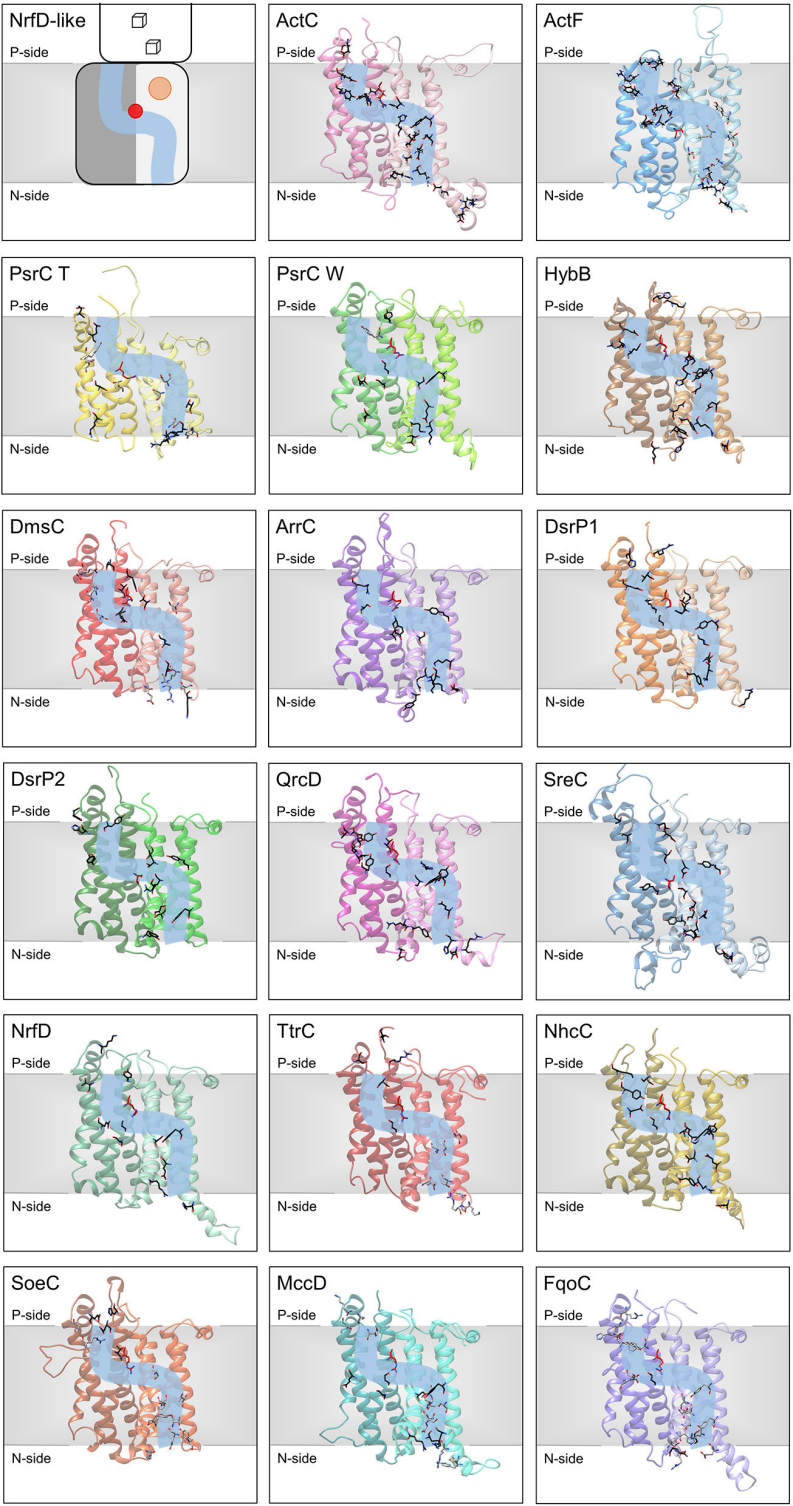
Ion-translocation pathway. Structural models of NrfD-like subunits with amino acid residues putatively involved in ion-translocation. The top left side panel contains a general schematic representation of NrfD-like subunits. Each protein is composed by eight common TMH that form two four-helix bundle (TMHs 1–4, light gray [lighter colors in the following panels) and TMHs 5–8, dark gray (darker colors in the following panels)] related by a 180° rotation around an axis perpendicular to the membrane. The light blue line schematizes the ion-conducting pathway and the red dot indicates the presence of the arginine residue (Arg395^ActC^), possibly acting as the gate of the pathway. The orange circle points to the quinone/quinol-binding site, which is in close proximity to the [4Fe-4S]^+2/+1^ cluster ([3Fe-4S]^1+/0^ in ActC) (cube) present at the peripheral subunit. The other panels contain the structural models obtained for the 8 common TMHs, the amino acid residues, putatively involved ion-pathways are indicated and depicted as sticks along the light blue line and are highlighted in black (conserved residues in respective sequence alignments), gray (amino acid residues present in the model able to conduct protons, but not conserved in respective sequence alignments) and red (equivalent to Arg395^ActC^). Please note that all models are oriented with the P-side of the membrane at the top of each panel, except for SoeC that is oriented with the N-side of the membrane at the top of the respective panel (see more in text and in legend of Figure 6).

In *R. marinus* ActC and ActF subunits, the putative ion-conducting pathway was suggested to be formed by two half-channels: a N-side half-channel in the first four-helix bundle (TMH 1–4) and a P-side half-channel in the second four-helix bundle (TMH 5–8) (Sousa et al., 2018). ActC and ActF ion-conducting pathways are composed of conserved amino acid residues within all ACIII complexes (Sousa et al., 2018) (Figure 7, ActC; Table 2).

We were able to identify, in all NrfD-like subunits, amino acid residues that may constitute a N-side half-channel in the first four-helix bundle (TMH 1–4) and a P-side half-channel in the second four-helix bundle (TMH 5–8). The amino acid residues that compose these putative ion-conducting pathways are conserved within each subunit: ActC, ActF, HybB, ArrC, DsrP, QrcD, SreC, NrfD and NhcC subunits (Figure 7, Table 2). However, in PsrC, DmsC, TtrC, SoeC, MccD and FqoC subunits, the two half-channels were not so easy to define, as we observed less conserved residues.

In *T. thermophilus* PsrC structure, the putative ion-conducting pathway was suggested to be formed by a N-side half-channel in the second four-helix bundle (TMH 5–8) and a P-side half-channel in the first four-helix bundle (TMH 1–4) (Jormakka et al., 2008). Of the proposed residues in the N-side half-channel at TMH 5-8 (Glu224^PsrC_T^, Thr220^PsrC_T^, Arg177^PsrC_T^) (Jormakka et al., 2008), only Arg177^PsrC_T^ residue is conserved among other PsrC T subunits. Mutation of Asp218^PsrC_W^, the equivalent to Arg177^PsrC_T^ in *W. succinogenes* PsrC, and Ser192^PsrC_W^ residues, both located at TMH 5-8 in the N-side half-channel, resulted in inhibition or reduction of polysulfide respiration, respectively (Jormakka et al., 2008). Nevertheless, we identified conserved amino acid residues (Figure 7, PsrC T; Table 2) that could form two half-channels, resembling those identified in ActC and ActF subunits. Our identification is supported by substitution studies of amino acid residues located at TMH 1–4 on our proposed N-side half-channel (Tyr106^PsrC_W^ and Glu146^PsrC_W^) and located at TMH 5-8 on the P-side half-channel (Glu225^PsrC_W^, Ser185^PsrC_W^, Ser188^PsrC_W^ and Tyr310^PsrC_W^) of *W. succinogenes* PsrC model, resulted in strains with a compromised polysulfide respiration (Dietrich and Klimmek, 2002). These data suggest those residues may be important for proton translocation, since PsrABC catalyzes an endergonic reaction dependent of electrochemical potential (Dietrich and Klimmek, 2002; Calisto et al., 2021).

The presence of an ion-conducting pathway in HybB is also supported by the observations that replacement of Arg89^HybB^, Tyr99^HybB^, Glu148^HybB^ and His184^HybB^ in *E. coli* HybB (N-side half-channel TMH 5–8) significantly decreased hydrogen oxidation (Lubek et al., 2019). Addition of a protonophore increases hydrogen oxidation in these mutated strains (Lubek et al., 2019), showing that ion translocation and catalytic activity are coupled.

Previously for QrcD, only the N-side half-channel, present in the first four-helix bundle (TMH 1–4) as observed for ActC and ActF subunits, was proposed to be present (Duarte et al., 2018). This N-side half-channel was hypothesized to translocate protons from the N-side of the membrane to the quinone-binding site, for quinone reduction (Duarte et al., 2018). However, we identified conserved amino acid residues that could be part of a P-side half-channel in the second four-helix bundle (TMH 5–8) (Figure 7, QrcD; Table 2).

Although the ion-conducting pathways present in the different NrfD-like subunits are not composed by the same amino acid residues, they are localized at the same spatial position. Noticeably, we identified a conserved arginine residue (Arg395^ActC^, Arg239^PsrC_T^, Arg305^PsrC_W^, Arg329^HybB^, Arg271^DmsC^, Arg324^ArrC^, Arg358^QrcD^, Arg306^NrfD^, Arg280^TtrC^, Arg345^NhcC^, Arg297^SoeC^, Arg306^MccD^, Arg293^FqoC^) located in middle of the membrane (in TMH 8) in a position that coincides with that at which the two proton half-channels converge (Figure 7). In *Desulfovibrio vulgaris* DsrP this is occupied by a lysine (Lys329^DsrP1^). We hypothesized that the residue at this position may perform a gate keeping role for proton translocation across the membrane. In fact, water molecules around Arg239^PsrC_T^ were observed in the structure of PsrC from *T. thermophilus* (Jormakka et al., 2008) and mutation of Arg305^PsrC_W^ resulted in inhibition of polysulfide respiration (Dietrich and Klimmek, 2002). Although, we hypothesized a relevant function for this arginine (or Lys329^DsrP1^) residue, it is not conserved in ActF, DsrP2 and SreC. In these subunits the gating role may be performed by a conserved serine residue (Ser238^ActF^, Ser204^DsrP2^ and Ser235^SreC^), which is located in middle of the membrane in TMH 5, also coinciding with the convergence of the two half-channels (Figure 7).

The members of the NrfD family are transmembrane proteins (8 to 10 TMHs) characterized by the presence of structural repeats, composed of two four-helix bundles, harboring ion-translocation pathways and a quinone/quinol-binding site.

The quinone/quinol-binding site of NrfD-like subunits is located at the P-side of the membrane in the first four-helix bundle (TMHs 1–4), always in vicinity of the peripheral iron-sulfur subunit. The peripheral subunits of SoeABC complex are hypothesized to be located at the N-side (Dahl et al., 2013; Boughanemi et al., 2020) and thus SoeC would be expected to be in an inverted orientation in the membrane when comparing to the other NrfD-like proteins. In this way, its quinone-binding site would be present on the N-side of the membrane. We identified, in TMH 2 close to the entry of the quinone/quinol pocket, a serine residue (Ser164^ActC^) that appears to be important for interaction with quinone/quinol molecule, since this serine residue is strictly conserved in all NrfD-like subunits that interact with quinone/quinol.

Our structural models reinforce the possible existence of ion-translocation pathways in all NrfD-like subunits and the contribution of the NrfD-like subunit-containing complexes to energy transduction. NrfD-like subunit-containing complexes may perform energy transduction by an indirect-coupling mechanism and may generate or consume electrochemical potential (Calisto et al., 2021). *W. succinogenes* PsrABC activity was shown to be dependent on electrochemical potential (Dietrich and Klimmek, 2002), while QrcABCD activity was shown to be coupled to the formation of electrochemical potential (Duarte et al., 2018). The ion-translocation pathways, observed in NrfD-like subunits, are formed by amino acid residues that may establish hydrogen bonds, allowing proton translocation by a Grotthuss-type mechanism (de Grotthuss, 1806; Cukierman, 2006). A semiconserved arginine amino acid residue, located in the middle of the membrane at the intersection of the two ion half-channels, was here suggested to play an important role as gate keeper in ion-translocation. In ActF, DsrP2 and SreC the gating role may be performed by a conserved serine.

The data here presented indicate that NrfD-like subunits are possibly the ion translocating modules of different enzymes, involved in a vast array of metabolisms, such as the oxygen, nitrogen, sulfur, arsenate and hydrogen cycles. All NrfD-like subunits containing complexes may be thus energy transducing membrane machines that contribute to energy conservation in vast range of organisms and under multiple growth conditions.

## Materials and Methods

The selection of the complete NrfD-like subunits dataset was performed using protein BLAST (pBLAST) analysis tool (default parameters) running at KEGG's (Kyoto Encyclopedia of Genes and Genomes) database, which only contains data on fully sequenced genomes (Ogata et al., 1999; Kanehisa et al., 2006, 2008). The information used in this study was that available by October 2020.

Protein search was performed using the amino acid sequences of ActC and ActF from *R. marinus* (rmr:rmar_0223, rmr:rmar_0226), PsrC from *T. thermophilus* (tth:tt_c0153) and *W. succinogenes* (wsu:ws0118), NrfD (eco:b4073), DmsC (eco:b0896), and HybB (eco:b2995) from *E. coli*, TtrC from *S. enterica* (sty:sty1737), SreC from *A. ambivalens* (aamb:d1866_07880), SoeC from *A. aeolicus* (aae:aq_1231), QrcD (dvu:dvu0692) and DsrP (dvu:dvu1286) from *D. vulgaris*, NhcC from *D. desulfuricans* (dds:ddes_2040), ArrC from *A. ehrlichii* (aeh:mlg_0214) and MccD from *W. succinogenes* (wsu:ws0382). Results with *e*-value <0.01 were accepted for further analysis (ca. 4,545 protein sequences).

The 4,545 amino acid sequences were clustered according to their identity using the CD-HIT tool (50% identity) (Huang et al., 2010). The resulting 551 representative sequences were aligned using PROMALS3D (standard parameters, using *R. marinus* ActC as structural template, PDB: 6F04) (Pei et al., 2008). The dendrogram was constructed using RAxML toll and the Neighbor-Joining (NJ) method at the CIPRES gateway portal (Miller et al., 2010). The obtained dendrogram was visualized and manipulated in Dendroscope (Huson et al., 2007).

To generate the structural models of NrfD-like subunits we used the amino acid sequence from *W. succinogenes* PsrC (wsu:ws0118), *E. coli* NrfD (eco:b4073), *E. coli* DmsC (eco:b0896), *E. coli* HybB (eco:b2995), *S. enterica* TtrC (sty:sty1737), *A. ambivalens* SreC (aamb:d1866_07880), *A. aeolicus* SoeC (aae:aq_1231), *D. vulgaris* QrcD (dvu:dvu0692), *D. vulgaris* DsrP1 (dvu:dvu1286), *Allochromatium vinosum* DsrP2 (alv:alvin_1262), *D. desulfuricans* NhcC (dds:ddes_2040), *A. ehrlichii* ArrC (aeh:mlg_0214), *W. succinogenes* MccD (wsu:ws0382) and *C. tepidum* FqoC (cte:ct0494) subunits. We developed the structural models using Phyre2 for predicting protein structure by homology modeling under intensive mode, and ActC *R. marinus* (PDB: 6F0K2) structure was selected as template (Kelley et al., 2015; Sousa et al., 2018). Protein structural model visualization and figure construction were performed using Chimera (Pettersen et al., 2004).

Quinone/quinol-binding sites and ion-translocation pathways were identified by sequence conservation analysis, after declustering the amino acid sequences from dendrogram branches, selected as containing ActC, ActF, PsrC, NrfD, DmsC, HybB, TtrC, SreC, SoeC, QrcD, DsrP, NhcC, ArrC, MccD and FqoC sequences. Sequence alignments were performed using PROMALS3D for each dendrogram group and visualized using JalView software (Waterhouse et al., 2009).

## Data Availability Statement

The raw data supporting the conclusions of this article will be made available by the authors, without undue reservation.

## Author Contributions

FC and MP conceived the study, analyzed the results, and wrote the manuscript. All authors contributed to the article and approved the submitted version.

## Conflict of Interest

The authors declare that the research was conducted in the absence of any commercial or financial relationships that could be construed as a potential conflict of interest.
